# Targeting HSP90 with Ganetespib to Induce CDK1 Degradation and Promote Cell Death in Hepatoblastoma

**DOI:** 10.3390/cancers17081341

**Published:** 2025-04-16

**Authors:** Lea Amelie Jung, Salih Demir, Alina Hotes, Eiso Hiyama, Tomoro Hishiki, Emilie Indersie, Sophie Branchereau, Stefano Cairo, Roland Kappler

**Affiliations:** 1 Department of Pediatric Surgery, Dr. von Hauner Children’s Hospital, LMU University Hospital, 80337 Munich, Germany; jung.lea@campus.lmu.de (L.A.J.); salih.demir@med.uni-muenchen.de (S.D.); alina.hotes@med.uni-muenchen.de (A.H.); 2 Natural Science Center for Basic Research and Development, Hiroshima University, Hiroshima 739-0046, Japan; eiso@hiroshima-u.ac.jp; 3 Department of Pediatric Surgery, Graduate School of Medicine, Chiba University, Chiba 260-8677, Japan; hishiki@chiba-u.jp; 4 XenTech, 91000 Evry, France; emilie.indersie@xentech.eu; 5 Bicêtre Hospital, AP-HP Paris Saclay University, 94270 Paris, France; sophie.branchereau@aphp.fr; 6 Champions Oncology, Inc., Rockville, MD 20850, USA; scairo@championsoncology.com; 7 Pediatric Research Institute (IRP), 35127 Padova, Italy

**Keywords:** hepatoblastoma, HSP90, ganetespib, CDK1, apoptosis

## Abstract

Background/Objectives: Hepatoblastoma, the most common malignant liver tumor in pediatric patients, is characterized by a remarkably low mutation rate, thereby impeding targeted therapies. Current treatment regimens rely on conventional cytotoxic agents that often cause severe adverse effects, necessitating the search for novel, less toxic therapeutic approaches. Methods: In this study, we explored the anti-tumor potential of heat shock protein 90 (HSP90) inhibitors using a unique collection of hepatoblastoma in vitro models. Results: Among the five tested inhibitors, we identified ganetespib as the most effective, significantly suppressing tumor cell growth while sparing healthy, non-tumor cells. Ganetespib treatment at low nanomolar concentrations markedly reduced cell proliferation, impaired long-term survival, and inhibited three-dimensional spheroid growth, ultimately leading to the induction of apoptosis. Mechanistically, ganetespib downregulated the expression of the HSP90 client protein cyclin-dependent kinase 1, a key cell cycle regulator controlling G2/M phase transition, which is heavily upregulated in hepatoblastoma. This disruption consequently resulted in cell cycle arrest, further contributing to its anti-tumor effects. Conclusions: HSP90 inhibition by ganetespib demonstrates significant potential as a novel therapeutic strategy for hepatoblastoma, offering a potential alternative to current cytotoxic treatments with fewer adverse effects.

## Simple Summary

Hepatoblastoma is the most common pediatric liver cancer. Current treatments use strong drugs that can have serious side effects, so safer options are needed. In this study, we tested a group of drugs called HSP90 inhibitors, which work by blocking the proteins that cancer cells need to grow. Out of the five drugs we tested, one called ganetespib showed the best results. It was very effective at stopping the cancer cells from growing but had little impact on healthy cells. Even at low doses, ganetespib slowed down cancer cell growth, reduced their ability to survive over time, and caused the cancer cells to die. It works by disrupting a key protein named CDK1 that helps cancer cells divide. Our findings suggest that ganetespib could be a promising new treatment for hepatoblastoma, potentially offering a safer and less toxic alternative to current therapies.

## 1. Introduction

Hepatoblastoma (HB), though rare, is the most common malignant liver tumor in pediatric patients, with an incidence of 0.5–1.5 cases per million children, typically presenting within the first three years of life [1]. Generally, HB has a favorable long-term survival outcome of over 80% [2]. This has been achieved by the treatment of standard-risk patients with cisplatin monotherapy and using a combination regimen involving dose-dense cisplatin and doxorubicin for high-risk patients [3,4]. However, these chemotherapeutic agents show a variety of significant side effects, ranging from reversible symptoms such as nausea and vomiting to severe toxicities, including ototoxicity and nephrotoxicity [5,6]. Consequently, identifying alternative or adjunct therapies is critical to reducing the treatment burden and improving patient outcomes.

HB is characterized by a very low mutation rate [7], with comprehensive sequencing studies identifying only a very limited number of recurrent mutations, each occurring in subsets of patients [8,9]. Approximately two-thirds of HB cases harbor mutations in components of the Wnt signaling pathway, specifically in the β-catenin (CTNNB1) [10] or adenomatous polyposis coli (APC) [11] genes. Notably, the druggability of the Wnt signaling pathway has been challenging in the clinical setting [12]. Mutations of the nuclear factor erythroid-derived 2-like (NFE2L2) gene and in the telomerase reverse transcriptase (TERT) promoter have been detected only in 6% of HB cases [13]. As targeted therapies rely on the presence of specific, actionable mutations, identifying non-mutational dependencies, such as overexpressed proteins, might lead to the identification of molecular vulnerabilities in HB.

Heat shock protein 90 (HSP90) is a highly conserved and ubiquitous chaperone protein that functions as a homodimer, binding to so-called client proteins and facilitating their maturation into functionally active forms [14]. With several hundred proteins relying on HSP90, it serves as a key modulator of important processes that are integral to the hallmarks of cancer [15]. Consequently, HSP90 overexpression has been observed in a variety of cancers [16], including liver cancer [17]. Notably, HSP90 expression is increased along with the stepwise progression of hepatocarcinogenesis from low-grade dysplastic nodules to high-grade hepatocellular carcinomas and is associated with prognostic factors such as poorer histological grade of differentiation, microvascular invasion, and intrahepatic metastasis [18]. Evidence supporting the relevance of HSP90 in HB includes elevated HSP90 mRNA levels in genetically engineered HB mouse models [19] and preliminary in vitro studies using tanespimycin on patient-derived xenograft cell culture models [20]. The therapeutic potential of HSP90 inhibition has been demonstrated in several human cancers, including a phase II study of tanespimycin in breast cancer [21] and the approval of pimitespib for gastrointestinal stromal tumors that progress after chemotherapy [22]. Moreover, ganetespib showed a manageable safety profile in patients with advanced HCC [23]. Collectively, these data suggest that HSP90 inhibition could represent a potential therapeutic option for HB patients.

This study aimed to investigate the in vitro effects of five HSP90 inhibitors on HB cell lines and to evaluate the most effective candidate, ganetespib, in the preclinical setting.

## 2. Materials and Methods

### 2.1. Cell Lines

The HB cell lines HepG2 (American Type Culture Collection, Manassas, VA, USA), HepT1 (from Dr. T. Pietsch), HuH6 (Japanese Collection of Research Bioresources, Osaka, Japan), the hepatocellular carcinoma cell lines Hep3B (American Type Culture Collection) and HUH7 (Japanese Collection of Research Bioresources), and the patient-derived xenograft cultures PDX214, PDX229, PDX233, PDX243, PDX282, PDX293B, PDX295, PDX303, PDX305, PDX310, PDX344, and PDX364 (XenTech, Evry, France) were used. As healthy controls, the human dermal fibroblasts HDFa (adult) and HDFn (neonatal), the embryonic kidney cell line HEK293 (all American Type Culture Collection), as well as the keratinocyte cell line HaCaT (CLS Cell Lines Service, Eppelheim, Germany) were used. The established tumor cell lines were cultured in RPMI 1640 containing 10% fetal bovine serum and 1% penicillin/streptomycin, the PDX cell lines in advanced DMEM/F12 with 10% fetal bovine serum, 1% penicillin/streptomycin, 1% L-glutamine (all from Thermo Fisher, Waltham, MA, USA), and 20 µM Y-27632 (Selleckchem, Chesterbrook, PA, USA), and the healthy controls in DMEM with 10% fetal bovine serum and 1% penicillin/streptomycin.

### 2.2. Western Blot Assay

Whole-cell lysates were collected in cell lysis buffer containing 30 mM TrisHCl/150 mM NaCl/1% Triton-X/10% glycerol/5 mM PMSF/0.5 M DTT/0.5 M Na_2_VO_4_/10 mM β-glycerophosphate/500 mM sodium fluoride/1:100 Halt^TM^ protease and phosphatase inhibitor (Thermo Fischer). Denatured proteins were separated in Novex^TM^ 4–20% gradient gel (Thermo Fischer) and transferred by Trans-Blot Turbo (Bio-Rad, San Francisco Bay Area, CA, USA) using a transfer cassette. Membranes were washed and blocked with PBS-T and 5% milk (Roth, Gießen, Germany) in PBS-T. The membranes were incubated at 4 °C overnight with the following primary antibodies: anti-HSP90 (Cell Signaling, Danvers, MA, USA) 1:1000, anti-CDC2 (Cell Signaling) 1:1000, anti-PLK1 (Cell Signaling) 1:1000, anti-AURKA (Cell Signaling) 1:1000, anti-alpha-tubulin (Sigma-Aldrich, St. Louis, MO, USA) 1:4000, anti-GAPDH (Thermo Fischer) 1:10,000, and anti-beta-actin (Cell Signaling) 1:2000 diluted in 5% milk in PBS-T. After washing, membranes were incubated with appropriate secondary antibodies: goat anti-mouse immunoglobulin HRP (Dako, Santa Clara, CA, USA) 1:1000 and anti-rabbit igG HRP-linked antibody (Cell Signaling) 1:10,000 in 5% milk in PBS-T. Protein detection was performed using enhanced chemiluminescence ECL reagent (Cytiva, Marlborough, MA, USA) with the ChemiDoc XRS + imaging system (Bio-Rad). The density of protein bands was measured with the ImageJ (version 1.54d) image processing tool.

### 2.3. Cell Viability Assay

MTT (3-(4,5-dimethylthiazol-2-yl)-2,5-diphenyltetrazoliumbromide) assays were performed on 5 × 10^5^ cells per well in a 96-well-plate after treating cells for 48 h with ten increasing doses ranging from 5 nM to 100 µM of carboplatin, cisplatin, and doxorubicin as well as the HSP90 inhibitors geldanamycin, luminespib, pimitespib, tanespimicin, and ganetespib (all from Selleck Chemicals GmbH, Houston, TX, USA) as described earlier [24]. The viability of cells was measured after 48 h treatment by reading absorbance on a sunrise plate reader (Tecan, Männedorf, Switzerland). Area under the curve (AUC) was calculated using GraphPad Prism 8 (GraphPad Software, San Diego, CA, USA), and overall effectiveness was calculated as a Z-score using the formula: [(Individual AUC value)-(mean AUC)]/standard deviation. Therapeutic window scores (TWS) were calculated by using the formula: (mean AUC of healthy controls)/(mean AUC of tumor lines).

### 2.4. RNA Expression Analysis

RNA expression data (GSE131329) of 14 non-cancerous liver and 53 HB tissues were downloaded from the R2 genomics analysis and visualization platform on 16 November 2023 (http://r2.amc.nl) and used for gene set enrichment analysis (GSEA). Enriched pathways were detected by using the Enrichr web tool (https://maayanlab.cloud/Enrichr/), and enrichment plots were created by utilizing the Molecular Signature Database (MSigDB) (https://www.gsea-msigdb.org) both accessed on 18 December 2024. Candidate gene expression levels were compared between non-cancerous liver and tumor samples using an unpaired *t*-test, and *p* < 0.05 was a cutoff for significance.

Quantitative gene expression analysis of ganetespib (5 nM) and DMSO-treated cells was accomplished using total RNA according to procedures described earlier [25]. The following primer pairs (5′->3′orientation) were used: CDK1, GCATCCCATGTCAAAAACTTGG, TTCAGTGCCATTTTGCCAGAA; PLK1, GGTTTTCGATTGCTCCCAGC, GGGGGTTCTCCAAGCCTTTA; AURKA, GAAGACTTGGGTCCTTGGGT, AAATATCCCCGCACTCTGGC; and TBP, GCCCGAAACGCCGAATAT, CCGTGGTTCGTGGCTCTCT. Data were normalized to the expression level of the housekeeping gene TATA-Box-binding-Protein (TBP). Relative RNA expression was calculated using the ∆∆CT method and expressed as fold-change relative to the corresponding DMSO-treated sample [26].

### 2.5. Proliferation Assay

Cell proliferation was measured using the Click-iT EdU Imaging Kit (Thermo Fischer) according to the manufacturer’s instructions. A total of 1 × 10^5^ cells were seeded in a 24-well plate and incubated for 24 h. Cells were then labeled with the EdU working solution and treated with either 5 nM ganetespib or DMSO for 24 h. Cells were then fixed with 3.7% paraformaldehyde, permeabilized with 0.5% Triton-X in PBS, and finally stained with Hoechst 33342 (Thermo Fischer) and the Click-iT staining cocktail containing the Click-iT reaction buffer, CuSO_4_, and Alexa Fluor acid (Thermo Fischer). Images were taken with the EVOS M7000 system (Thermo Fischer). Relative proliferation was calculated by referring the EdU-positive proliferating cells to the total Hoechst 33342-positive cells.

### 2.6. Colony Formation Assay

A total of 2 × 10^3^ cells were seeded in a 6-well plate and after 72 h, they were treated with either 5 nM ganetespib or DMSO. The treatment was refreshed after another 72 h, and cells were incubated for a total of 9 days. Images were taken at a 4× magnification using the EVOS M7000 system (Thermo Fischer).

### 2.7. Spheroid Assay

A total of 1 × 10^3^ cells were seeded in a 96 ultra-low attachment well plate (Corning Incorporated, Corning, NY, USA) and incubated for 72 h. Images were taken of the formed spheroids and then again after treatment with 5 nM of ganetespib or DMSO for 72 h. Both imaging and size measuring of spheroids on days 0 and 3 were performed by using the EVOS M7000 system (Thermo Fischer). The spheroid volume was calculated for 0 h and 72 h by the formula: [(length (µm) × width µm)^2^)/2].

### 2.8. Live and Dead Staining

Treated spheroids were stained with 1 µM Calcein-AM (Biolegend, Hercules, CA, USA) and 1 mg/mL propidium iodide (Sigma-Aldrich) in serum-free medium (Thermo Fisher) for 20 min at 37 °C. Images were taken with the EVOS M7000 system (Thermo Fischer).

### 2.9. Cell Cycle Analysis

For synchronization, cells were cultured in a serum-free starvation medium for 48 h. The degree of synchronization was validated after 24 h using the Click-iT EdU Imaging Kit (Thermo Fischer) and after 48 h using standard immunofluorescent detection of the proliferation marker Ki-67 [27] and flow cytometry (Supplemental Figure S1). Then, serum-free medium was replaced with normal media and the cells were exposed to DMSO or 10 and 50 nM of ganetespib for 48 h. Cells were harvested and fixed overnight with 80% ice-cold ethanol at −20 °C. The next day, 5 × 10^5^ cells were incubated with 300 µL cell cycle staining buffer, consisting of 50 μg/mL propidium iodide (Sigma-Aldrich), 0.5% Triton-X-100 (Sigma-Aldrich) and 2 µg/mL RNAse (Qiagen, Hilden, Germany) in dH_2_O, in the dark at 37 °C for 1 h. The distribution of cell cycle phases was measured by the LSRFortessa cell analyzer (BD Biosciences, East Rutherford, NJ, USA), and data analysis was conducted by FlowJo software v10.4 (BD Biosciences), applying the Watson Pragmatic model.

### 2.10. Apoptosis Assay

A total of 1 × 10^5^ cells were seeded in a 24-well plate and incubated for 24 h. Cells were then treated with 5 nM ganetespib or DMSO for 24 h. Cells were then stained with Hoechst 33342 and the cell event caspase 3/7 detection reagent (both Thermo Fischer). Imaging was accomplished with the EVOS M7000 system (Thermo Fischer). Relative apoptosis was calculated by referring to the apoptotic caspase 3/7-positive cells to the total Hoechst 33342-positive cells.

### 2.11. Statistical Analyses

Data were visualized, showing the mean ± standard deviation (SD) or standard error of the mean (SEM), and analyzed by GraphPad Prism 8 (GraphPad Software). For the comparison of the two conditions, statistical significance was determined by Student’s *t*-test. For Kaplan–Maier survival curves, Mantel–Cox log-rank analysis was applied. For all the statistical models, the significance was considered for values *p <* 0.05.

## 3. Results

### 3.1. Ganetespib Is the Most Effective HSP90 Inhibitor for Impeding HB Viability

**Figure 1:**
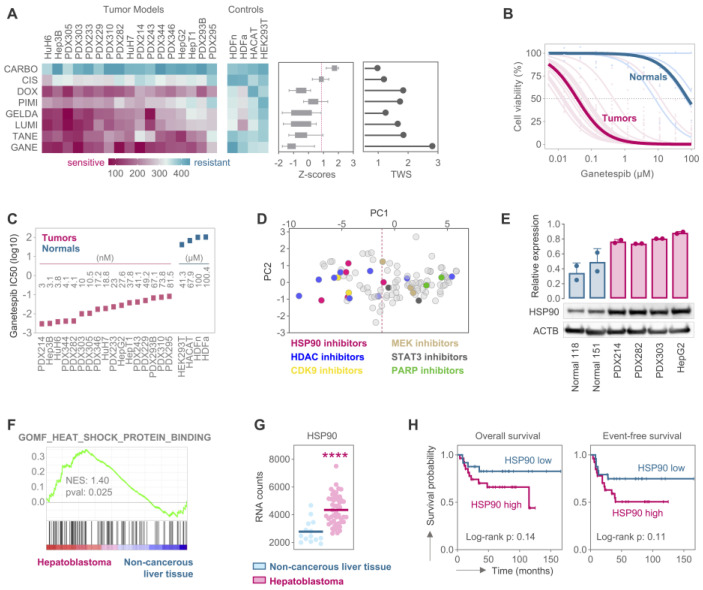
(**A**) A heat map showing the drug responses of 17 liver tumor models and 4 non-cancerous controls towards 5 HSP90 inhibitors (pimi)tespib, (gelda)namycin, (lumi)nespib, (tane)spimicin, and (gane)tespib as well as the standard-of-care drugs (carbo)platin, (cis)platin, and (dox)orubicin. Corresponding Z-scores and therapeutic window scores (TWS) are depicted as box-with-whiskers and lollipop plots, respectively. (**B**) Viability curves of liver tumor models towards 10 increasing concentrations of ganetespib (5 nm–100 µM). Dark pink and blue curves represent the mean response of the tumor models and healthy controls, respectively. (**C**) IC50 values of ganetespib-treated cell lines. (**D**) Principal component analysis of 83 compounds in our in vitro drug-testing platform. The drugs positioned on the right-hand side of the dashed line, which represents the PC1 of cisplatin, were considered ineffective. (**E**) Western blot analysis showing the HSP90 protein expression levels in two normal liver tissues and four hepatoblastoma models. Beta actin (ACTB) was used as a loading control. Bar graph shows the mean relative band density of two independent experiments ± SD. (**F**) Enrichment plot of gene ontology obtained from gene set enrichment analysis (GSEA) of 53 HB patients versus 14 non-cancerous livers from the publicly available GSE131329 data set. (**G**) Comparative RNA expression analysis of HSP90 in 53 HB patients and 14 non-cancerous liver samples from the GSE131329 data set, utilizing R2 Genomics Analysis and Visualization Platform. (**H**) Kaplan-Meier curves displaying overall survival (OS) and event-free survival (EFS) probabilities for patients with HB of the GSE131329 data set with either high (n = 27) or low (n = 25) HSP90 expression. Median expression was considered as cutout, and the log-rank Mantel–Cox test was used to calculate significance. **** *p* < 0.0001.

In order to determine the therapeutic potential of HSP90 inhibition in HB patients, we tested a panel of HSP90 inhibitors—geldanamycin, luminespib, pimitespib, tanespimicin, and ganetespib—across 10 increasing concentrations on a comprehensive set of conventional liver tumor cell lines, patient-derived xenograft cultures, and healthy control cell lines using MTT-based cell viability assays. All HSP90 inhibitors demonstrated potent growth inhibitory effects on tumor cells while leaving normal cells almost unaffected (Figure 1A, left). Compared to conventional chemotherapy that is given to HB patients, such as carboplatin and cisplatin, all HSP90 inhibitors showed superior efficacy. Notably, most of the inhibitors even outperformed doxorubicin, a treatment reserved for high-risk HB patients [28]. Among the tested drugs, ganetespib emerged as the most effective, exhibiting remarkably high tumor-suppressive activity across all HB models. This was reflected in its smaller area under the curve (AUC) values and low Z-scores (Figure 1A, left and middle). Furthermore, ganetespib displayed the highest therapeutic window score, indicating its potential for flexible dose adjustments in the clinical setting (Figure 1A, right). The comparison of the drug inhibition curves between tumor models and healthy cells, as well as the corresponding half-maximal inhibitory concentrations (IC50), highlighted a >1000-fold difference in response, confirming the strong growth inhibitory effect of ganetespib with minimal toxicity to normal cells at comparable concentrations (Figure 1B,C).

We then integrated the response data of the HSP90 inhibitors into our recently published drug testing platform containing response data for 78 drugs [24]. By performing principal component analysis on the AUC values, we discovered HSP90 inhibitors as one of the most effective and tumor-suppressing drug categories, grouping them alongside HDAC and CDK9 inhibitors (Figure 1D). These categories demonstrated significantly greater efficacy compared to cisplatin, used as a reference drug.

To investigate the underlying mechanism of the HB-suppressive effects of HSP90 inhibitors, we examined the expression of HSP90 at the protein level. Western blot analysis of two normal liver tissues and four HB models revealed a pronounced overexpression of HSP90 in HB compared to mild expression observed in normal liver tissues (Figure 1E, Figure S2). Additionally, gene set enrichment analysis of publicly available RNA sequencing data from 53 HB patients and 14 non-cancerous liver samples (GSE131329) showed that the gene ontology term “heat shock protein binding” was significantly enriched in HB samples (Figure 1F). Moreover, HSP90 RNA levels were dramatically upregulated in HB samples (Figure 1G), and patients with high HSP90 expression showed a trend towards poor overall and event-free survival (Figure 1H).

Together, these findings suggest that elevated HSP90 levels and increased heat shock protein binding render HB highly susceptible to HSP90 inhibition, resulting in suppressed cell growth.

### 3.2. Ganetespib Significantly Suppresses HB Growth Potential

**Figure 2:**
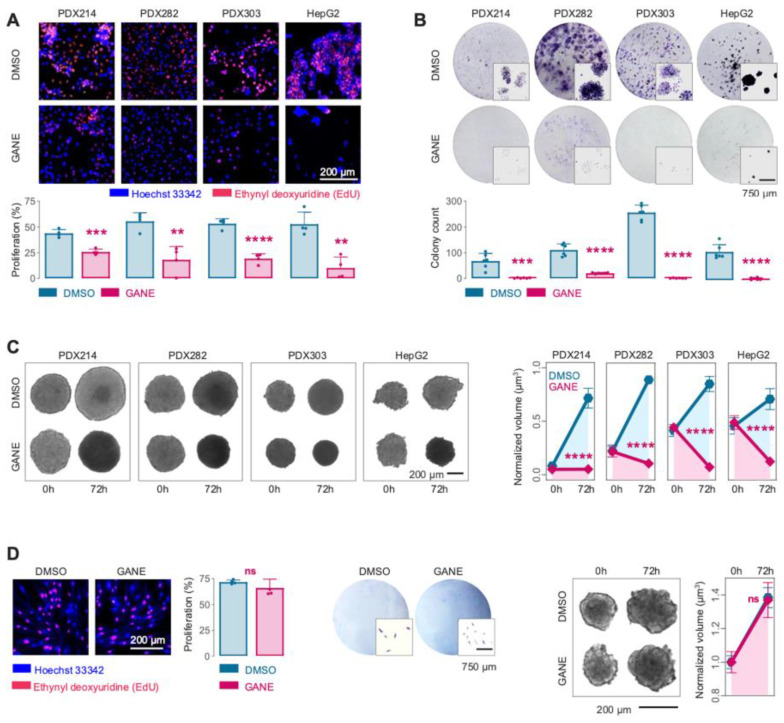
(**A**) Immunofluorescence images (top) and corresponding calculation of the proliferating cells (red) in relation to total cells (blue) upon DMSO or 5 nM (gane)tespib exposure for 24 h. (**B**) Crystal violet stainings of formed colonies (top) and absolute colony counts (bottom) are demonstrated upon DMSO or 5 nM (gane)tespib treatment for 10 days. (**C**) HB spheroids images and respective normalized spheroid volumes were displayed following DMSO or 5 nM (gane)tespib exposure for 72 h. (**D**) Proliferation, colony formation, and spheroid assays as in A-C on neonatal fibroblasts. Bar and line graphs represent the mean ± SEM of two independent experiments. Statistics were calculated using a two-tailed unpaired Student’s *t*-test, with ** *p* < 0.01, *** *p* < 0.001, **** *p* < 0.0001, ns = not significant.

To evaluate the impact of ganetespib, the most effective HSP90 inhibitor, on the growth properties of HB, we treated four high-risk HB models with the drug at IC50 concentrations and assessed growth-related features using multiple approaches. Proliferative activity of tumor cells, detected by the incorporation of nucleoside analogs into newly synthesized DNA of actively dividing cells, was markedly inhibited after 24 h of ganetespib treatment (Figure 2A).

Furthermore, the long-term survival potential of HB cells was significantly reduced, as evidenced by fewer and smaller colonies formed over a 10-day period in ganetespib-treated wells (Figure 2B). Notably, in a three-dimensional spheroid model, ganetespib treatment resulted in substantially smaller spheroid volumes, indicating compromised cell viability and growth (Figure 2C). In contrast, normal fibroblasts maintained their proliferative capacity in response to ganetespib, although they did not form colonies (Figure 2D). These results underscore the potent ability of ganetespib to hinder the growth and survival of HB cells across different experimental models while sparing normal cells.

### 3.3. Ganetespib Triggers Tumor Cell Death in HB Models

**Figure 3:**
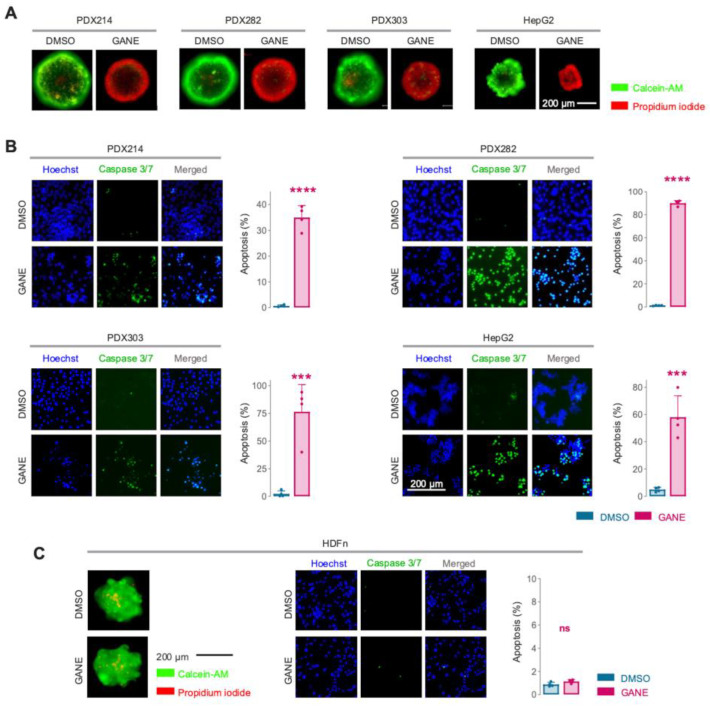
(**A**) Microscopy images of three-dimensional hepatoblastoma spheroids upon 72 h exposure to DMSO or 5 nM (gane)tespib. Cell death was detected by live (Calcein-AM, green) and dead (propidium iodide, red) staining. (**B**) Portions of apoptotic cells were determined by active caspase 3/7 staining after 24 h treatment with DMSO or 5 nM (gane)tespib. (**C**) Cell death and caspase activation assays as in (**A**,**B**) on neonatal fibroblasts. Bar graphs represent the mean ± SEM of two independent experiments with duplicates. Statistics were calculated using a two-tailed unpaired Student’s *t*-test, with *** *p* < 0.001, **** *p* < 0.0001, ns = not significant.

Treatment of HB spheroids with ganetespib not only inhibited growth but also led to volume shrinkage, a hallmark of cell death in three-dimensional models. To confirm this observation, we performed live and death staining on ganetespib-treated spheroids. As anticipated, a 72 h exposure to ganetespib resulted in a significant increase in propidium iodide-positive cells, particularly in the central and proliferative zones of the spheroids (Figure 3A). Additionally, all HB models treated with ganetespib for 24 h showed significantly higher levels of active caspase 3 and 7 substrates, highlighting the induction of apoptosis (Figure 3B). Of note, normal fibroblasts were unaffected by ganetespib treatment (Figure 3C). These findings collectively demonstrate ganetespib’s ability to induce selective tumor cell death through activation of caspase-dependent apoptotic pathways.

### 3.4. Elevated Expression of the HSP90 Client CDK1 Drives Hyperactivation of Cell Cycle Mechanisms in HB

**Figure 4:**
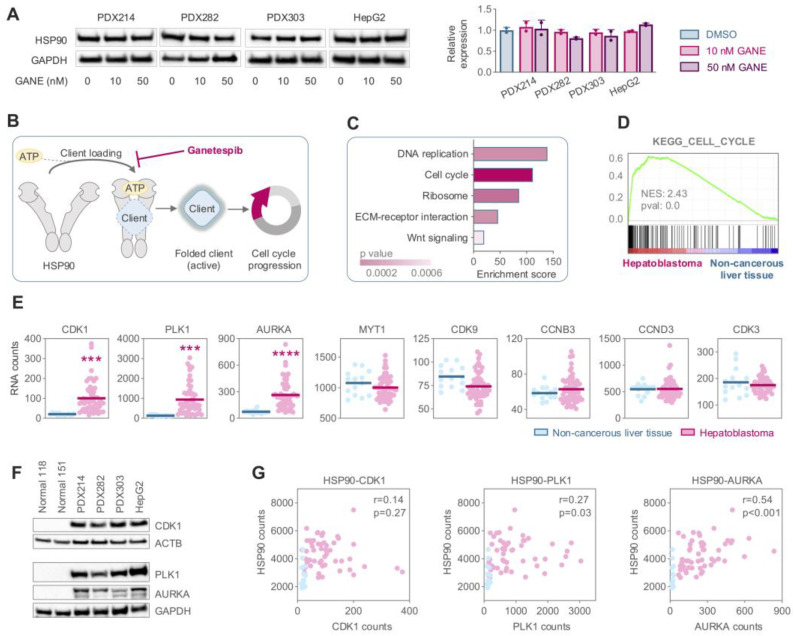
(**A**) Western blot analysis (left) and the bar graphs with corresponding density calculation of the bands (right) showing alterations in heat shock protein 90 (HSP90) protein levels upon increasing concentrations of (gane)tespib for 24 h. Glycerinaldehyd-3-phosphat-dehydrogenase (GAPDH) was used as loading control. (**B**) Schematic illustration of recruitment of a client by HSP90. (**C**) KEGG pathway enrichment scores of 767 significantly upregulated (*p* < 0.05, fold > 2) genes from RNA sequencing analysis of GSE131329 data set. (**D**) Enrichment plot showing the KEGG category cell cycle, obtained from gene set enrichment analysis (GSEA) of the GSE131329 data set. (**E**) Comparative RNA expression analysis of HSP90 clients from the GSE131329 data set, utilizing R2 Genomics Analysis and Visualization Platform. Statistics were calculated using a two-tailed unpaired Student’s *t*-test, with *** *p* < 0.001, **** *p* < 0.0001. (**F**) Western blot showing the expression of cyclin-dependent kinase 1 (CDK1), polo-like kinase 1 (PLK1), and aurora kinase A (AURKA) in non-cancerous liver tissue and hepatoblastoma models. Beta actin (ACTB) and GAPDH served as loading controls. (**G**) Correlation of gene expression between CDK1, PLK1, and AURKA with HSP90 of the GSE131329 data. Two-tailed Pearson test was performed, and correlation coefficient was calculated.

HSP90 inhibition is known to disrupt interactions between HSP90 and its client proteins [14]. To investigate this mechanism in HB, we first examined the impact of ganetespib exposure on HSP90 protein expression. Western blot analysis revealed no significant changes in HSP90 expression levels when HB cells were treated with 10 or 50 nM ganetespib (Figure 4A, Figure S2). This suggests that ganetespib does not induce degradation of HSP90 itself but likely exerts its effect by preventing ATP-dependent binding of HSP90 to its client proteins (Figure 4B). To identify HSP90 client proteins implicated in HB, we conducted KEGG pathway analysis on 767 upregulated genes (*p*-value < 0.05, fold change > 2) retrieved from publicly available RNA sequencing data (GSE131329) comparing 53 HB patients to 14 non-cancerous liver samples. This analysis highlighted “DNA replication” and “cell cycle” as the most significantly enriched terms (Figure 4C,D), consistent with the established role of numerous HSP90 clients in cell cycle regulation [29]. Utilizing the same RNA sequencing dataset, we further explored transcriptional changes in known HSP90 client proteins associated with the cell cycle [30]. Among these, cyclin-dependent kinase 1 (CDK1), polo-like kinase 1 (PLK1), and aurora kinase A (AURKA) exhibited dramatically increased transcriptional expression in HB patients compared to non-cancerous liver samples, suggesting a pivotal role as HSP90 clients in maintaining cell cycle activity in HB (Figure 4E). Protein-level analysis corroborated these findings, as all three candidate proteins were undetectable in non-cancerous liver tissues but highly expressed in HB models, as shown by Western blot analysis (Figure 4F, Figure S2). Notably, CDK1, PLK1, and AURKA showed strong co-expression with HSP90 in HB patients (Figure 4G). Collectively, these data confirm that cell cycle is profoundly activated in HB tumors, with elevated client protein levels as key drivers of dysregulated cell cycle regulation, potentially through their interaction with HSP90.

### 3.5. Ganetespib-Induced Inhibition CDK1 Leads to G2/M Arrest

**Figure 5:**
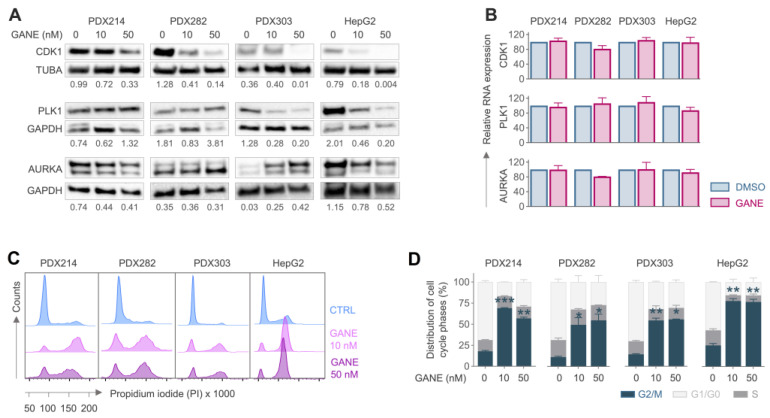
(**A**) Western blot and calculation of the normalized band densities demonstrate the expression of CDK1, PLK1, and AURKA proteins upon increasing concentrations of (gane)tespib for 24 h. Tubulin alpha 1a (TUBA) and glycerinaldehyd-3-phosphat-dehydrogenase (GAPDH) were used as loading controls. (**B**) PCR-based relative RNA expression analysis of gantespib treated tumor cells normalized to the housekeeping gene TBP. (**C**) Histograms showing the propidium iodide-labeled DNA distribution in synchronized hepatoblastoma models after 48 h treatment with DMSO, 10 nM or 50 nM (gane)tespib. (**D**) Stacked bar graphs displaying the distributions of cell cycle phases calculated according to the histograms. Significance was calculated in comparison to the DMSO control, using Student’s *t*-test with * *p* < 0.05, ** *p* < 0.01, *** *p* < 0.001.

Given the observed upregulation of several cell cycle-associated client proteins in HB, we wanted to investigate the functional consequences of their inhibition by ganetespib. When HB models were treated with increasing doses of ganetespib, CDK1 protein expression levels were drastically decreased (Figure 5A, Figure S2) while leaving its transcriptional levels almost unaffected (Figure 5B), suggesting that ganetespib prevents HSP90-mediated recruitment and stabilization of CDK1. In addition, we examined the two other HSP90 client proteins involved in the cell cycle and hepatoblastoma, namely the polo-like kinase 1 (PLK1) and the aurora kinase A (AURKA) [31,32]. However, ganetespib treatment reduced their protein levels in only two of the four HB models (Figure 5A, Figure S2), again with no consequences in RNA expression (Figure 5B). This indicates that while HSP90 inhibition principally affects both clients, they may not be the primary drivers of the dramatic tumor growth suppression and cell death observed across all HB models.

To investigate the impact of HSP90 inhibition on the cell cycle, we next synchronized HB models in the G0/G1 phase through serum starvation, as evidenced by the dramatic reduction in proliferating cells after 24 h, which became even more pronounced at 48 h (Supplemental Figure S1). Most importantly, subsequent ganetespib treatment caused significantly increased proportions of cells arrested in the G2/M phase of the cell cycle in all HB models after 48 h (Figure 5C,D), an effect already evident in fast-growing HepG2 and PDX303 cells after 24 h (Supplementary Figure S1D). This suggests that HSP90 inhibition by ganetespib leads to impairment of cell cycle progression by preventing the stabilization of CDK1, which is critical for G2/M transition.

Altogether, our data demonstrate that HSP90-mediated stabilization of CDK1 is an essential step for cell cycle progression and is hyperactivated in HB tumors. Moreover, we showed that ganetespib primarily targets CDK1 function, identifying CDK1 as a putative marker for response to ganetespib and the outcome of HB patients.

## 4. Discussion

The combination of cisplatin and doxorubicin has led to significant success in the treatment of HB [3,4]. However, these therapies are associated with severe adverse effects [33], underscoring the urgent need for novel, less toxic treatment options. In this study, we evaluated the efficacy of five HSP90 inhibitors in suppressing tumor growth using a comprehensive pediatric liver cancer testing platform. Our results showed that HSP90 is overexpressed in HB, positioning its inhibition as a promising target for cancer therapy. Ganetespib proved to be the most effective HSP90 inhibitor in HB models, exhibiting minimal impact on normal cells. We identified CDK1 as the key HSP90 client protein targeted by ganetespib in HB, leading to pronounced G2-M phase arrest and, ultimately, cell death.

Since the discovery of the first naturally occurring HSP90 inhibitor geldanamycin, HSP90 inhibition has been explored as an effective strategy for treating various malignancies, with 22 inhibitors tested in 186 cancer clinical trials [34]. Similarly, our in vitro screening with first-generation (geldanamycin), second-generation (tanespimycin and ganetespib), and third-generation (luminespib and pimitespib) HSP90 inhibitors demonstrated remarkable anti-tumor activity on HB models. Among the five tested inhibitors, ganetespib was the most promising candidate for the treatment of HB, showing potent growth inhibitory activity at very low nanomolar concentrations in all tested models while leaving normal cells nearly unaffected. Previous studies have shown the strong activity of ganetespib against various cancer types in the preclinical setting, including hepatocellular carcinoma [35]. More importantly, initial phase clinical trials in solid tumors showed that ganetespib is well tolerated [36]. Altogether, ganetespib demonstrates exceptional promise as a new therapy for HB, combining potent anti-tumor efficacy with minimal effects on normal cells. Coupled with its favorable safety profile in early-phase clinical trials, these findings position ganetespib as a leading candidate in the quest for safer and more precise therapies for HB.

HSP90 plays a key role in protein folding, facilitating the maturation of client proteins into their molecularly active conformations [14]. Proteomic studies have revealed more than 6000 HSP90 client proteins, including receptor tyrosine kinases, signaling proteins, transcription factors, and cell cycle regulatory proteins [37]. Malignant cells exploit the highly active HSP90 chaperone system to prevent misfolding and degradation of oncogenes and growth-promoting factors, leading to their constitutive activation [38]. Accordingly, elevated HSP90 expression is observed in the majority of cancer types, including liver cancer [17]. This dysregulation supports uncontrolled growth and disruption of cell cycle processes, a phenomenon underscored by the involvement of multiple HSP90 clients in cell signaling [29]. In line with these observations, our transcriptomic analysis of HB patients revealed gene signatures enriched for heat-shock protein binding and cell cycle pathways. One of the most important HSP90 clients as a key mediator of cell cycle progression is CDK1 [39]. Our investigation for relevant HSP90 clients revealed that CDK1 transcript levels are dramatically elevated in HB patients, while non-cancerous liver samples showed no detectable CDK1 protein expression. Overexpression of CDK1 has been detected in multiple cancer types, identifying it as a universal pan-cancer biomarker [40]. More importantly, survival analysis of 53 HB patients from the GSE131329 data set showed a clear association between high CDK1 expression and shorter survival periods. This is in line with previous work linking increased levels of CDK1 to inferior survival rates and unfavorable clinical outcomes, solidifying CDK1 as an important prognostic marker for many cancers [41]. Our data also indicated a massive decrease in CDK1 protein levels upon ganetespib treatment without altering its transcription, suggesting that CDK1 is post-translationally degraded due to blocking of HSP90 client recruiting activity. We further showed that ganetespib exposure led to G2/M arrest in all HB models, supporting the repression of CDK1 function upon ganetespib-mediated HSP90 inhibition, since CDK1 regulates transition between G2 phase and mitosis [42]. The downregulation of CDK1 as a consequence of exposure to the HSP90 inhibitors has been reported in different studies, such as by ganetespib in mantle cell lymphoma [43], tanespimycin in colon cancer [44], or by 17DMAG, a semi-synthetic derivative of geldanamycin in hepatocellular carcinoma [45].

One limitation of our study is that we do not provide direct evidence of a physical interaction between HSP90 and CDK1, nor confirm that HSP90 inhibition causes CDK1 proteasomal degradation. While our findings provide strong preclinical evidence supporting ganetespib as a potential therapy, additional studies are necessary to fully elucidate its mechanisms of action, particularly its effects on CDK1 and cell cycle progression.

Overall, these findings demonstrate the critical role of HSP90 in supporting cancer cell survival through its cell cycle-associated client proteins, particularly CDK1. Targeting HSP90 with inhibitors such as ganetespib offers a promising strategy for HB treatment, disrupting cancer’s dependency on HSP90 while inducing cell cycle arrest and apoptosis. Well-designed in vivo studies that closely mimic the clinical situation are essential to demonstrate the potential of HSP90 inhibition as a therapeutic option for HB.

## 5. Conclusions

This study highlights the potential of HSP90 inhibitors, particularly ganetespib, in suppressing HB tumor growth while sparing normal cells. HSP90 is overexpressed in HB and aids cancer cell survival by stabilizing proteins like CDK1, a critical regulator of cell cycle progression. Ganetespib effectively reduces CDK1 levels, induces G2-M arrest, and promotes cell death. With potent anti-tumor activity and tolerability in early clinical trials, ganetespib emerges as a promising candidate for HB therapy, targeting a crucial cancer dependency.

## Data Availability

No new data were created.

## Supplementary Materials

The following supporting information can be downloaded at https://www.mdpi.com/article/10.3390/cancers17081341/s1, Figure S1: Cell synchronization and cell cycle analysis, Figure S2: Original Western blot images.

## Abbreviations

The following abbreviations are used in this manuscript:

HB	Hepatoblastoma
HSP90	Heat shock protein 90
CDK1	Cyclin-dependent kinase 1
AUC	Area under the curve
TWS	Therapeutic window scores
GSEA	Gene set enrichment analysis
