# Supplementary material for: Targeting HSP90 with Ganetespib to Induce CDK1 Degradation and Promote Cell Death in Hepatoblastoma
Source: Cancers (Basel). 2025 Apr 16;17(8):1341. doi: 10.3390/cancers17081341 (PMC12026307; doi:10.3390/cancers17081341)
Supplement: Supplementary file 1 [file cancers-17-01341-s001.zip › cancers-3568989-supplementary.pdf]

Supplementary Figure 1: Cell synchronization and cell cycle analysis

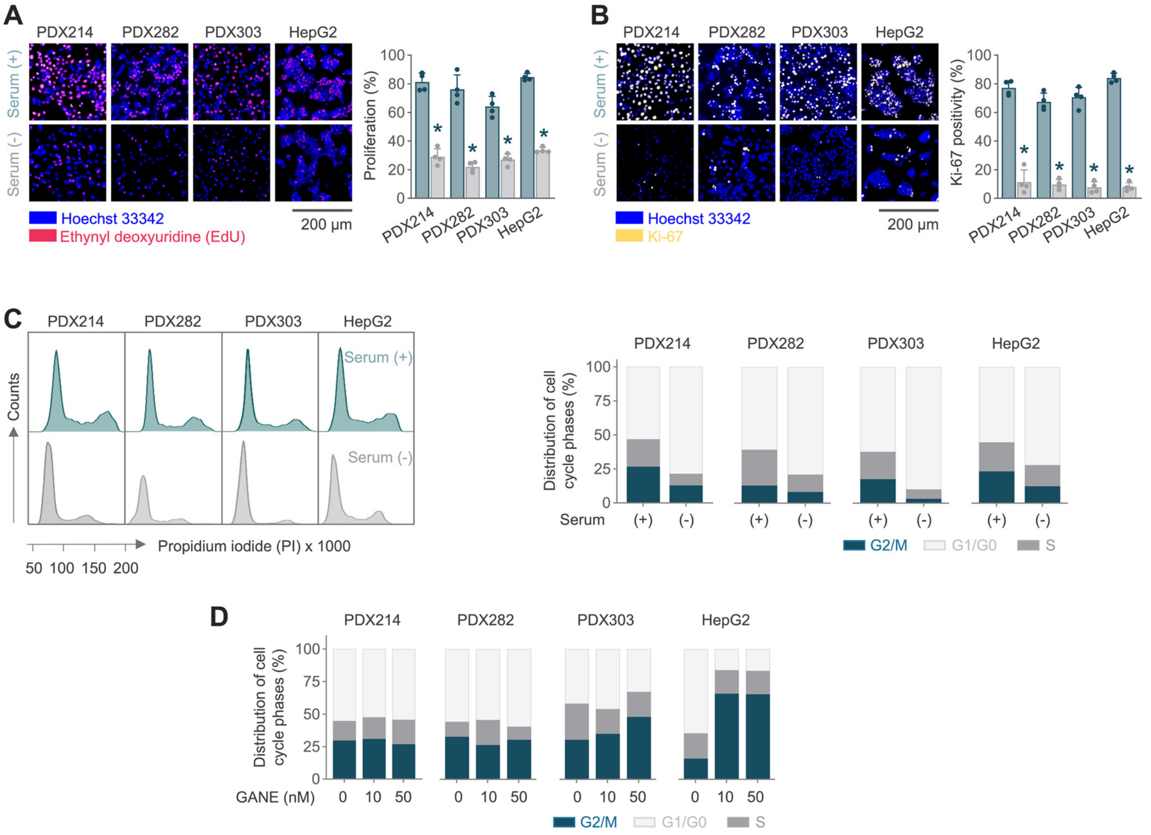

(A) Immunofluorescence images (left) and corresponding calculation (right) of proliferating cell populations (red) in relation to total nuclei (blue) upon 24h serum starvation. **(B)** Immunofluorescence images (left) and corresponding calculation (right) of proliferating Ki67 positive (yellow) in relation to total cells (blue) following 48h serum starvation **(C)** Histograms (left) depicting the propidium iodide-labeled DNA distribution in hepatoblastoma models incubated in either with serum or serum-free media for 48h. Corresponding stacked bar graphs (right) displaying the distributions of cell cycle phases in hepatoblastoma models in culturing media or 48h serum starvation. **(D)** Distributions of cell cycle phases in 48h serum starved-hepatoblastoma models upon 24h exposure to DMSO, 10 nM (gane)tespib or 50 nM (gane)tespib.

Supplementary Figure 2: Original Western blot images

Figure 1-E → HSP90 western blots

DISPLAY IMAGE

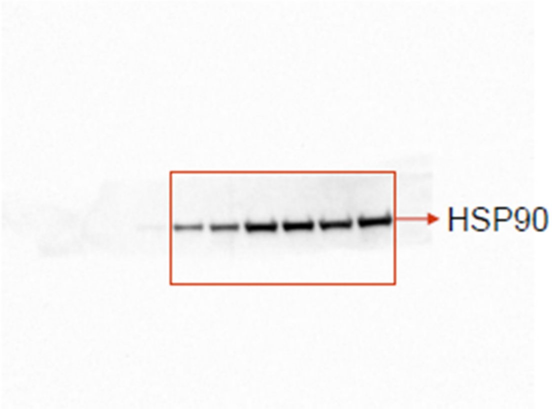

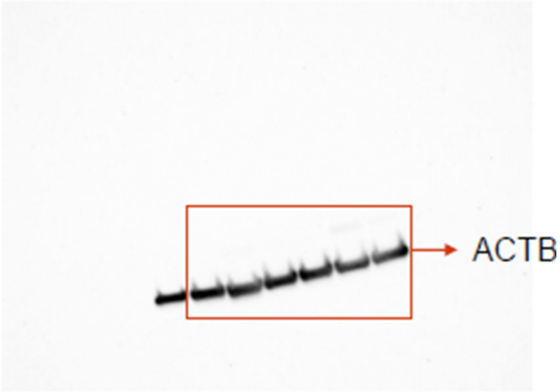

SECOND EXPERIMENT

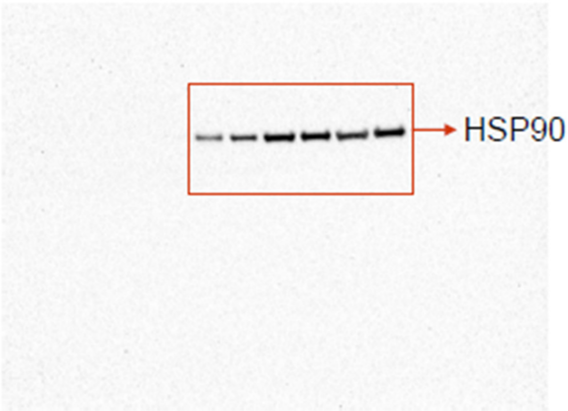

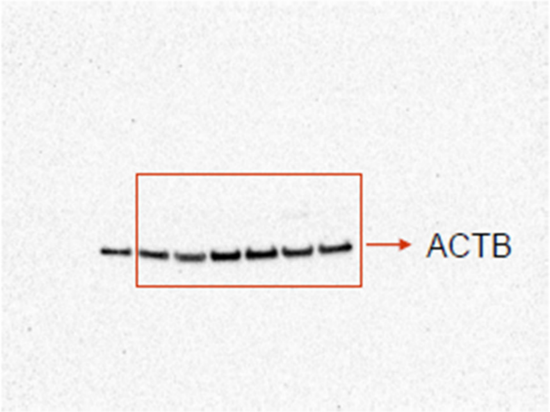

DISPLAY IMAGE- DENSITY

	HSP90	ACTB	EXPRESSION
Normal 118	9,620,288	38,376,877	0.2507
Normal 151	14,217,673	38,825,969	0.3662
PDX214	27,428,785	36,636,362	0.7487
PDX282	26,587,522	36,105,655	0.7364
PDX303	22,024,815	27,331,149	0.8059
HepG2	27,011,836	30,994,877	0.8715

SECOND EXPERIMENT-DENSITY

	HSP90	ACTB	EXPRESSION
Normal 118	7,652,782	17,415,723	0.4394
Normal 151	11,510,146	18,621,309	0.6181
PDX214	20,403,581	25,951,380	0.7862
PDX282	20,110,167	27,181,693	0.7398
PDX303	17,598,480	21,871,915	0.8046
HepG2	20,689,874	23,111,522	0.8952

Figure 4-A → HSP90 western blots (DISPLAY IMAGE)

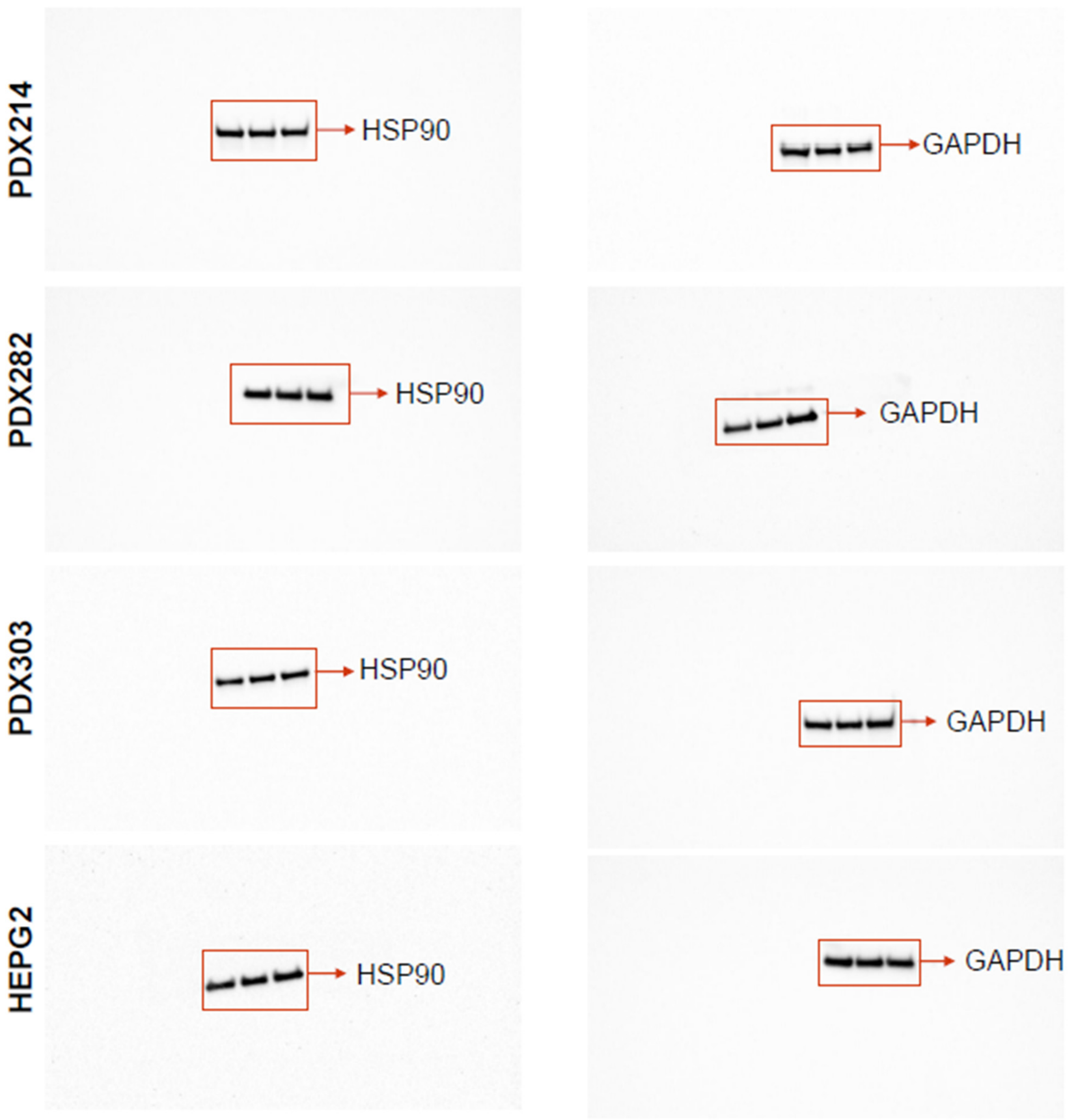

		HSP90	GAPDH	EXPRESSION
PDX214	DMSO	33,905,664	31,124,643	1.0894
	GANE 10 nM	33089250.00	30,989,492	1.0678
	Gane 50 nM	29959250.00	31,002,078	0.9664
PDX282	DMSO	23,186,300	20,018,815	1.1582
	GANE 10 nM	25,203,643	22,004,158	1.1454
	Gane 50 nM	30,165,785	33,070,179	0.9122

		HSP90	GAPDH	EXPRESSION
PDX303	DMSO	24,520,593	30,247,028	0.8107
	GANE 10 nM	26517158.00	32,863,978	0.8069
	Gane 50 nM	28742401.00	36,633,442	0.7846
HepG2	DMSO	25,787,865	32,109,765	0.8031
	GANE 10 nM	25,670,037	33,424,806	0.7680
	Gane 50 nM	28,327,714	30,285,149	0.9354

Figure 4-A → HSP90 western blots (SECOND EXPERIMENT)

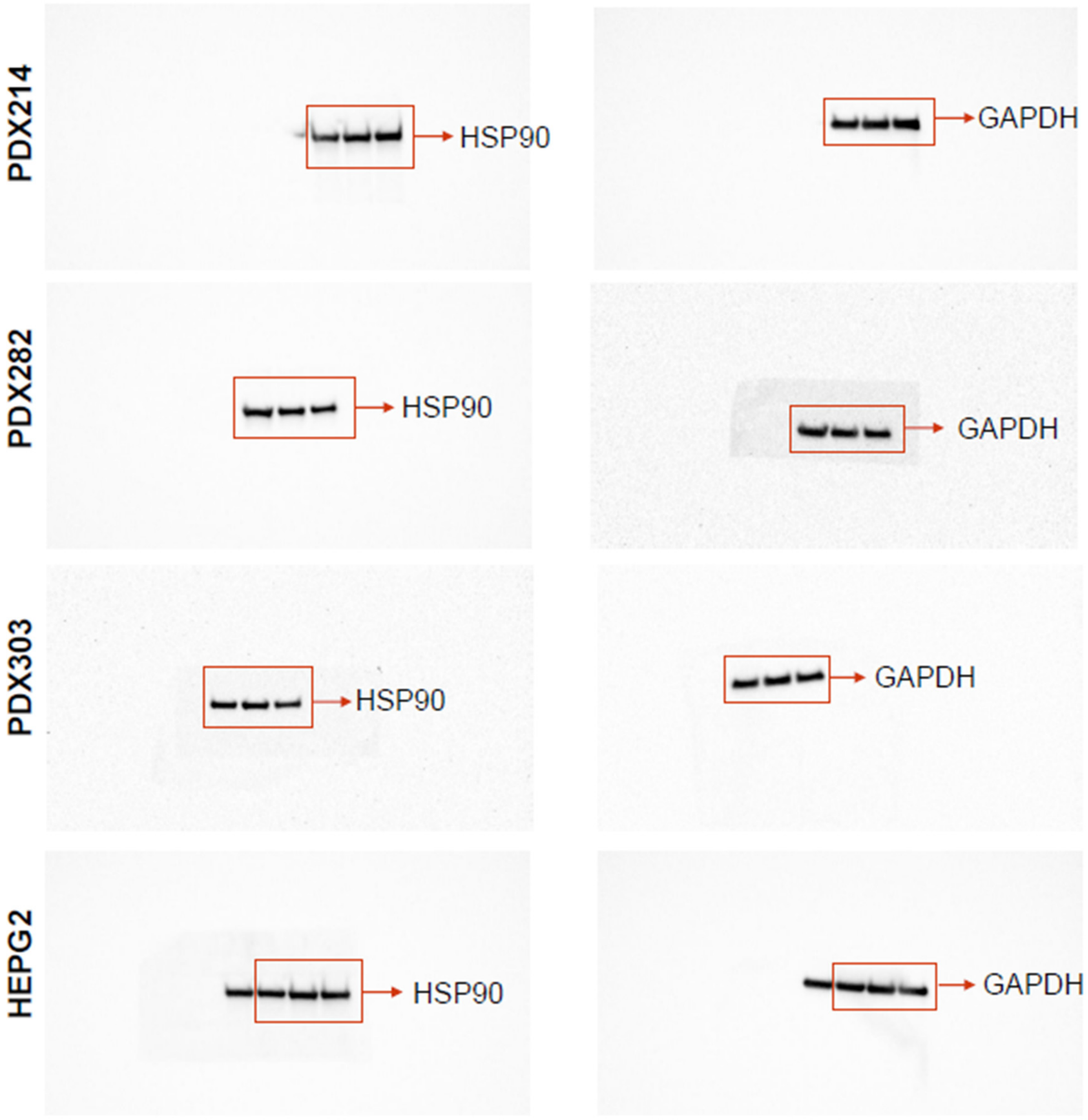

		HSP90	GAPDH	EXPRESSION
PDX214	DMSO	17,713,551	26,681,815	0.6639
	GANE 10 nM	22997137.00	29,323,250	0.7843
	Gane 50 nM	25541258.00	32,542,865	0.7848
PDX282	DMSO	26,494,593	30,925,321	0.8567
	GANE 10 nM	22,437,551	28,568,785	0.7854
	Gane 50 nM	19,115,723	26,743,057	0.7148

		HSP90	GAPDH	EXPRESSION
PDX303	DMSO	22,793,066	26,338,472	0.8654
	GANE 10 nM	24990723.00	32,077,179	0.7791
	Gane 50 nM	19051844.00	28,880,593	0.6597
HepG2	DMSO	19,711,359	28,174,865	0.6996
	GANE 10 nM	21,288,187	30,776,785	0.6917
	Gane 50 nM	19,683,238	25,372,401	0.7758

Figure 4-F → CDK1 western blots

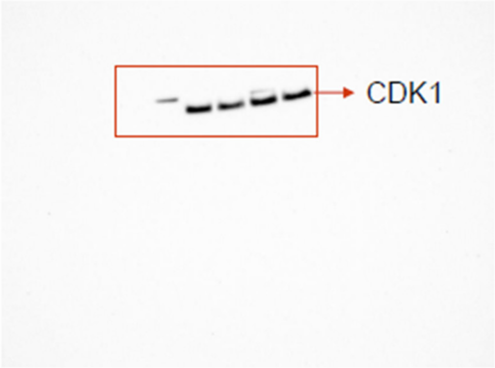

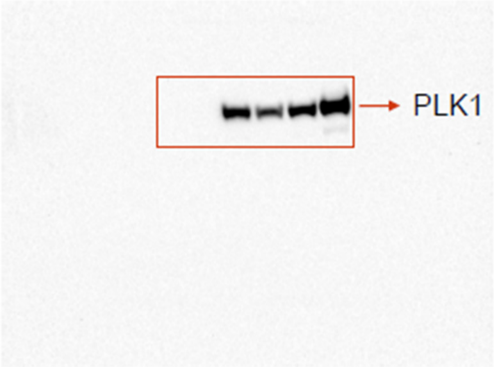

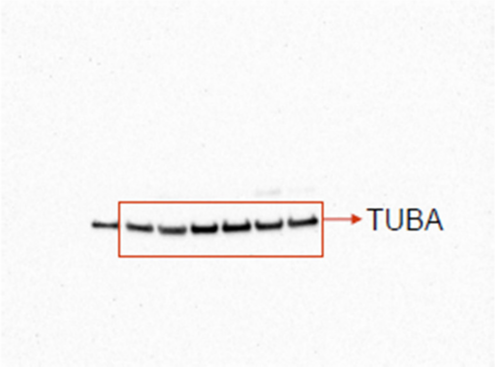

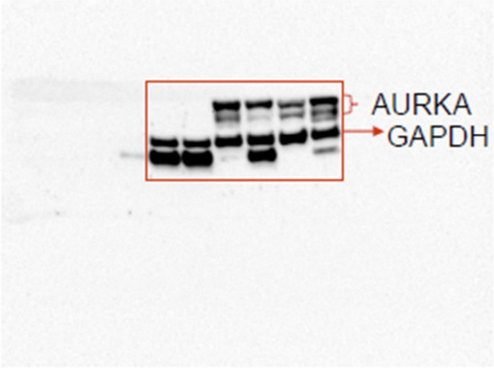

Figure 5-A → CDK1 western blots

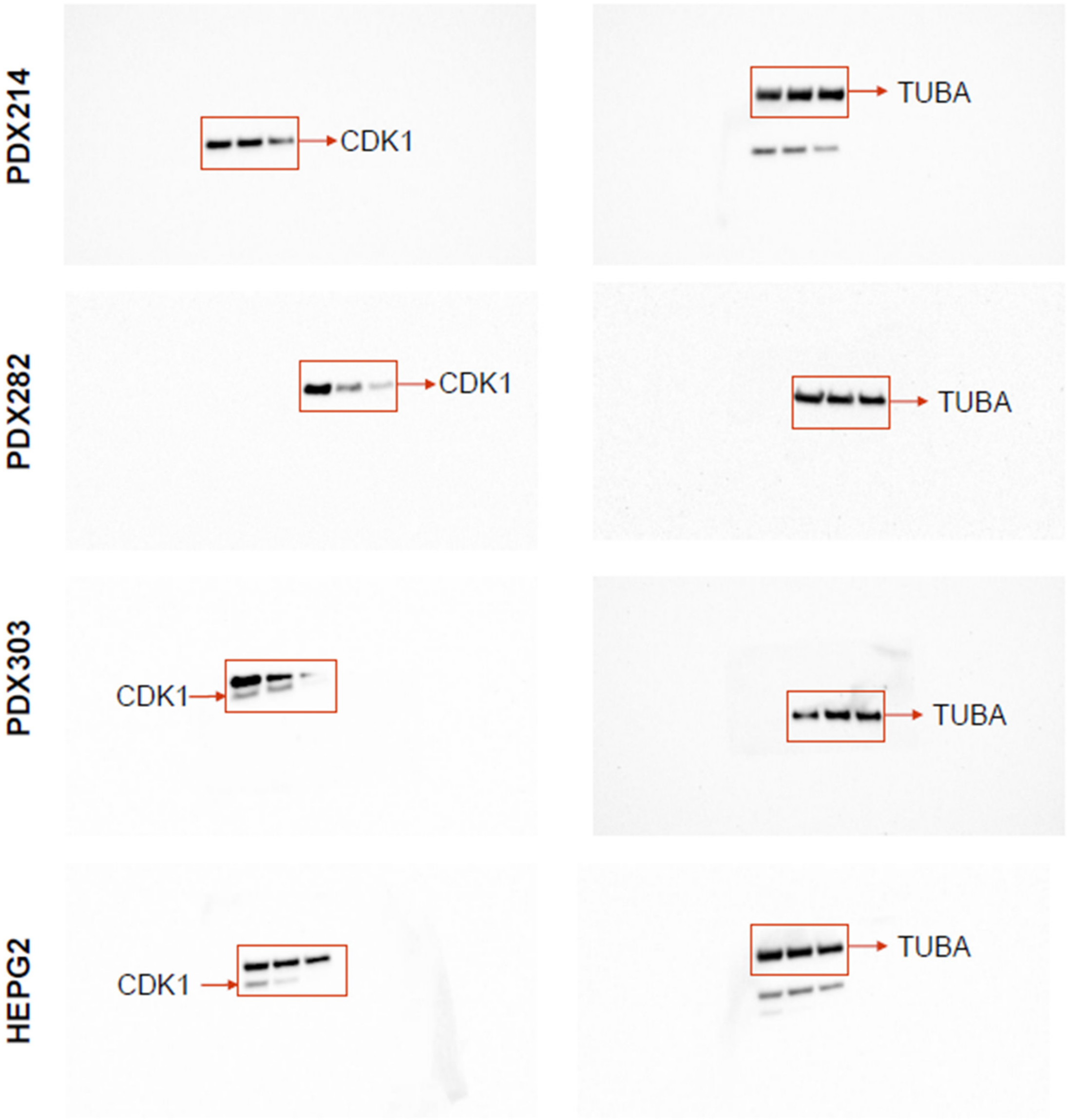

		CDK1	TUBA	EXPRESSION
PDX214	DMSO	32,693,300	33,034,170	0.9897
	GANE 10 nM	31121643.00	43,282,827	0.7190
	Gane 50 nM	15053037.00	44,960,877	0.3348
PDX282	DMSO	39,336,229	30,811,122	1.2767
	GANE 10 nM	11,581,137	27,758,715	0.4172
	Gane 50 nM	3,741,175	25,652,057	0.1458

		CDK1	TUBA	EXPRESSION
PDX303	DMSO	11,785,610	32,154,778	0.3665
	GANE 10 nM	12,178,681	30,623,176	0.3977
	Gane 50 nM	458,263	35,136,516	0.0130
HepG2	DMSO	31,659,865	39,950,413	0.7925
	GANE 10 nM	7,034,681	38,683,170	0.1819
	Gane 50 nM	161,950	32,726,099	0.0049

Figure 5-A → PLK1 western blots

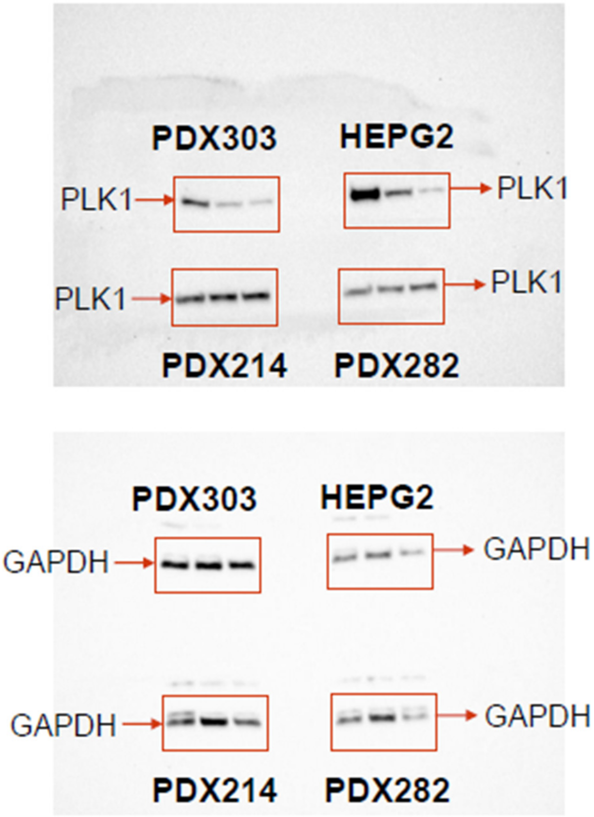

		PLK1	GAPDH	EXPRESSION
PDX214	DMSO	13,553.853	18,309.31	0.7403
	GANE 10 nM	16,311.581	26,327.82	0.6196
	Gane 50 nM	17,389.652	13,189.14	1.3185
PDX282	DMSO	16,968.329	9,366.15	1.8117
	GANE 10 nM	16,462.208	19,798.46	0.8315
	Gane 50 nM	19,060.359	5,008.22	3.8058

		PLK1	GAPDH	EXPRESSION
PDX303	DMSO	25,602.543	20,000.966	1.2801
	GANE 10 nM	6,506.338	23,621.016	0.2754
	Gane 50 nM	3,830.832	19,186.037	0.1997
HepG2	DMSO	36,682.158	18,242.765	2.0108
	GANE 10 nM	10,223.045	22,361.037	0.4572
	Gane 50 nM	1,764.669	8,768.602	0.2012

Figure 5-A → AURKA western blots

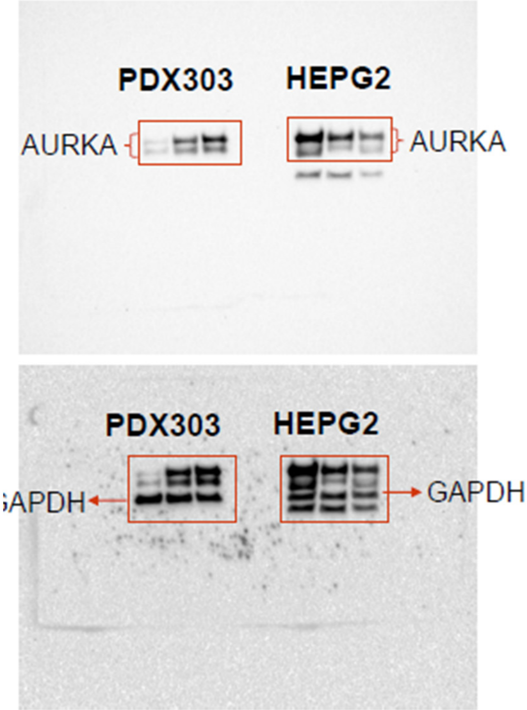

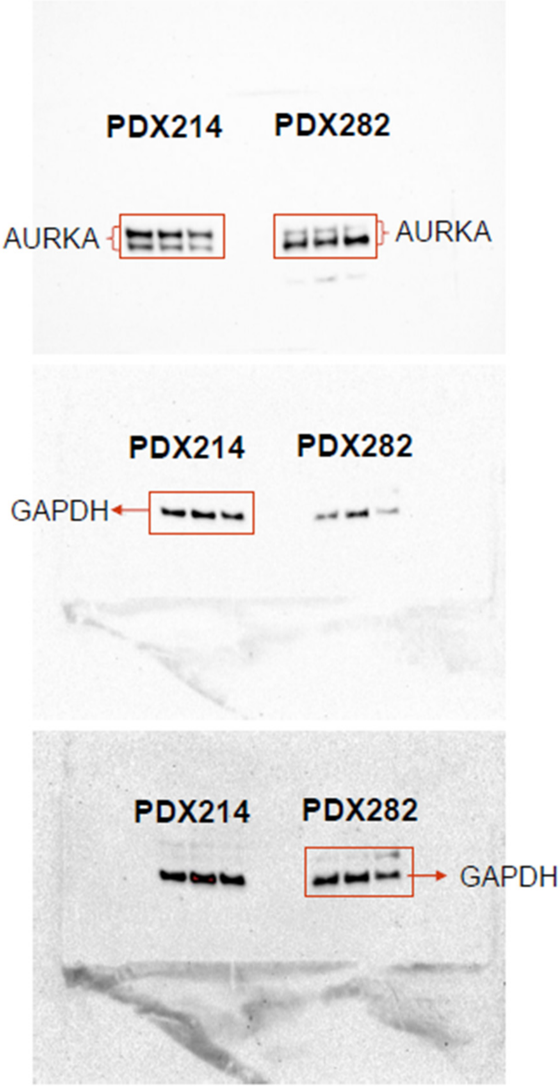

		AURKA	GAPDH	EXPRESSION
PDX214	DMSO	14,908.953	20,136.187	0.7404
	GANE 10 nM	11,484.660	25,914.844	0.4432
	Gane 50 nM	8,081.903	19,572.016	0.4129
PDX282	DMSO	1,397.719	3,997.102	0.3497
	GANE 10 nM	1,623.841	4,485.271	0.3620
	Gane 50 nM	727.920	2,378.549	0.3060
PDX303	DMSO	780.991	25,892.664	0.0302
	GANE 10 nM	5,725.033	22,743.250	0.2517
	Gane 50 nM	8,767.811	21,019.392	0.4171
HepG2	DMSO	21,753.631	18,878.602	1.1523
	GANE 10 nM	13,657.359	17,382.986	0.7857
	Gane 50 nM	7,684.752	14,566.279	0.5276
